# Hematology oncology practice in the Asia-Pacific APHCON survey results from the 6th international hematologic malignancies conference: bridging the gap 2015, Beijing, China

**DOI:** 10.18632/oncotarget.15655

**Published:** 2017-02-23

**Authors:** Xiao Jun Huang, Kaiyan Liu, David Ritchie, Borje Andersson, Jin Lu, Jian Hou, Adolfo de Fuente Burguera, JianXiang Wang, Allen Yeoh, Chenhua Yan, Daobin Zhou, Daryl Tan, Dong Wook Kim, Depei Wu, Elizabeth Shpall, Stephen Kornblau, Sattava Neelapu, Suradej Hongeng, Jianyong Li, Jiong Hu, Lian Sheng Zhang, Michael Wang, Pankaj Malhotra, Qian Jiang, Yazhen Qin, Raymond Wong, Richard Champlin, Frederick Hagemeister, Jason Westin, Swaminathan Iyer, Vikram Mathews, Yu Wang, Yu Hu, Zhijian Xiao, Zonghong Shao, Robert Z. Orlowski, Chor Sang Chim, Stephen Mulligan, Miguel Sanz, Keiya Ozawa, Simrit Parmar, Surapol Issaragrisil

**Affiliations:** ^1^ Peking University People’s Hospital, Peking University Institute of Hematology, Beijing, China; ^2^ Royal Melbourne Hospital, Melbourne, Australia; ^3^ MD Anderson Cancer Center, Houston, Texas, USA; ^4^ Shanghai Changzheng Hospital, Shanghai, China; ^5^ MD Anderson Cancer Center, Madrid, Spain; ^6^ Chinese Academy of Medical Sciences & Peking Union Medical College, Beijing, China; ^7^ Singapore General Hospital, Bukit Merah, Singapore; ^8^ Seoul St. Mary’s Hospital, S. Korea; ^9^ First Affiliated Hospital of Soochow University, Jiangsu Institute of Hematology, Jiangsu, China; ^10^ Ramathibodi Hospital, Bangkok, Thailand; ^11^ First Affiliated Hospital of Nanjing Medical University, Jiangsu Province Hospital, Nanjing, China; ^12^ Ruijin Hospital, Shanghai, China; ^13^ Gansu Provincial Key Laboratory of Hematology, Lanzhou, China; ^14^ Post Graduate Institute of Medical Education and Research, Chandigarh, India; ^15^ Prince of Wales Hospital, The Chinese University of Hong Kong, Hong Kong, China; ^16^ Methodist Hospital, Houston, Texas, USA; ^17^ Christian Medical College and Hospital, Vellore, India; ^18^ Wuhan Union Hospital, Wuhan, China; ^19^ Institute of Hematology and Hospital of Blood Diseases, Chinese Academy of Medical Sciences, Tianjin, China; ^20^ General Hospital of Tianjin Medical University, Tianjin, China; ^21^ Queen Mary Hospital, Hong Kong; ^22^ Royal North Shore Hospital, University of Sydney, Australia; ^23^ University Hospital La Fe, Valencia, Spain; ^24^ The Institute of Medical Science, University of Tokyo, Japan; ^25^ Faculty of Medicine Siriraj Hospital, Bangkok, Thailand; ^26^ National University Hospital, Singapore

**Keywords:** Asia, hematology, myeloma, leukemia, lymphoma

## Abstract

This report serves as a snapshot of the state-of-knowledge in the Asia Pacific (APAC) Hematology Oncology community, and establishes a baseline for longitudinal investigations to follow changes in best practices over time. The objective of this study was to understand the approach to hematologic diseases, common standards of care and best practices, issues that remain controversial or debated, and educational or resource gaps that warrant attention. We used mobile application to disseminate and distribute questionnaires to delegates during the 6^th^ international hematologic malignancies conference hosted by the APAC Hematology Consortium at Beijing, China. User responses were collected in an anonymous fashion. We report survey results in two ways: the overall responses, and responses as stratified between Chinese physicians and “Other” represented nationalities. Overall geographical concordance in survey responses was positive and strong. Perhaps more interesting than instances of absolute agreement, these data provide a unique opportunity to identify topics in which physician knowledge or opinions diverge. We assigned questions from all modules to broad categories of: patient information; diagnosis; treatment preference; transplantation; and general knowledge/opinion. On average, we observed a geographic difference of 15% for any particular answer choice, and this was fairly constant across survey modules. These results reveal utility and need for widespread and ongoing initiatives to assess knowledge and provide evidence-based education in real time. The data will be made more valuable by longitudinal participation, such that we can monitor changes in the state of the art over time.

## INTRODUCTION

In January 2015, the Asia-Pacific Hematology Consortium (APHCON) held sixth annual Bridging The Gap (BTG) conference series in Beijing, China where delegates participated in series of surveys using an innovative mobile education app called MDRing™. Results of the module on acute myeloid leukemia (AML) [1] and that of multiple myeloma (MM) [2] were published independently. These took a first pass at revealing common standards of care and best practices, issues that remain controversial or debated, and educational or resource gaps that warrant attention. Here we present highlights from the other 11 surveys (Table 1), and provide complete set of questions and answers as Supplemental Material. This report serves as a snapshot of state-of-knowledge in Asia Pacific hematology oncology community, and establishes baseline for longitudinal investigations to follow changes in best practices over time.

**Table 1:** BTG2015 survey module participation

Survey Module	Number of questions	Average response rate	Number of respondents / question		
			Overall	China	Other
Acute Lymphocytic Leukemia	13	87%	60 - 69	46 - 54	14 - 15
Acute Myeloid Leukemia	16	83%	84 - 105	68 - 86	15 - 19
Aplastic Anemia	12	88%	52 - 62	39 - 49	13
Cell Therapy	5	91%	59 - 62	46 - 49	12 - 13
Chronic Lymphocytic Leukemia	2	94%	36 - 37	21 - 22	15
Chronic Myeloid Leukemia	11	93%	53 - 58	41 - 46	12
Donor Selection	9	87%	60 - 68	45 - 53	14 - 15
Hodgkin's Lymphoma	6	90%	68 - 74	50 - 55	17 - 19
Non-Hodgkin's Lymphoma (part 1)	7	94%	63 - 67	47 - 51	16
Non-Hodgkin's Lymphoma (part 2)	7	66%	29 - 38	17 - 23	12 - 15
Multiple Myeloma	25	83%	53 - 76	40 - 61	13 - 15
Myelo- dysplastic/proliferative Neoplasms	12	86%	46 - 67	33 - 53	13 - 14
Thalassemia	6	87%	56 - 62	44 - 49	12 - 13

## MATERIALS AND METHODS

### Survey deployment and participation

We used the mobile application MDRing™ to conduct survey among delegates at the BTG 2015 Beijing conference. Survey consisted of 13 question and answer modules (Table 1). Average number of questions per module was 10, smallest included 2 questions, and the largest 25. Number of respondents ranged from 29 to 105, with an average of 64 respondents per question. The average per module response rate ranged from a low-end outlier 66% to 94%, with an average of 86%. This level of engagement far exceeds the minimal expectation of 60%, typically cited as sufficient for reporting.

### Data analysis

Data were initially analyzed as percentage of respondents selecting a particular answer. These values were calculated for the entire set of participants, and stratified based on national identity: China *versus* “Other”. We plotted every possible answer choice percentage for China *versus* Other and calculated coefficient of determination, or R^2^ value, as an overall assessment of agreement between geographic groups. For each question we used chi-squared analysis to determine whether observed answer choice percentages differed between geographies. We measured response concordance between geographic groups for each module in several ways. For each question we calculated R^2^, as above, as well as percent of multiple-choice answers selected in same rank order. We also determined range and mean difference in percent response for each answer choice, and the 95% confidence interval around that mean. Lastly, we grouped questions into five broad categories—diagnosis, stem cell transplant, general knowledge/opinion, patient info, and treatment preference—and estimated extent to which physicians agree with one another in each category. Degree of consensus is simply highest overall percent among a given question's multiple answer choices. That is, if choice A was selected by 75% of all respondents, maximum degree of consensus for that question was considered 75%.

## RESULTS and DISCUSSION

### Geographic stratification

**Figure 1:**
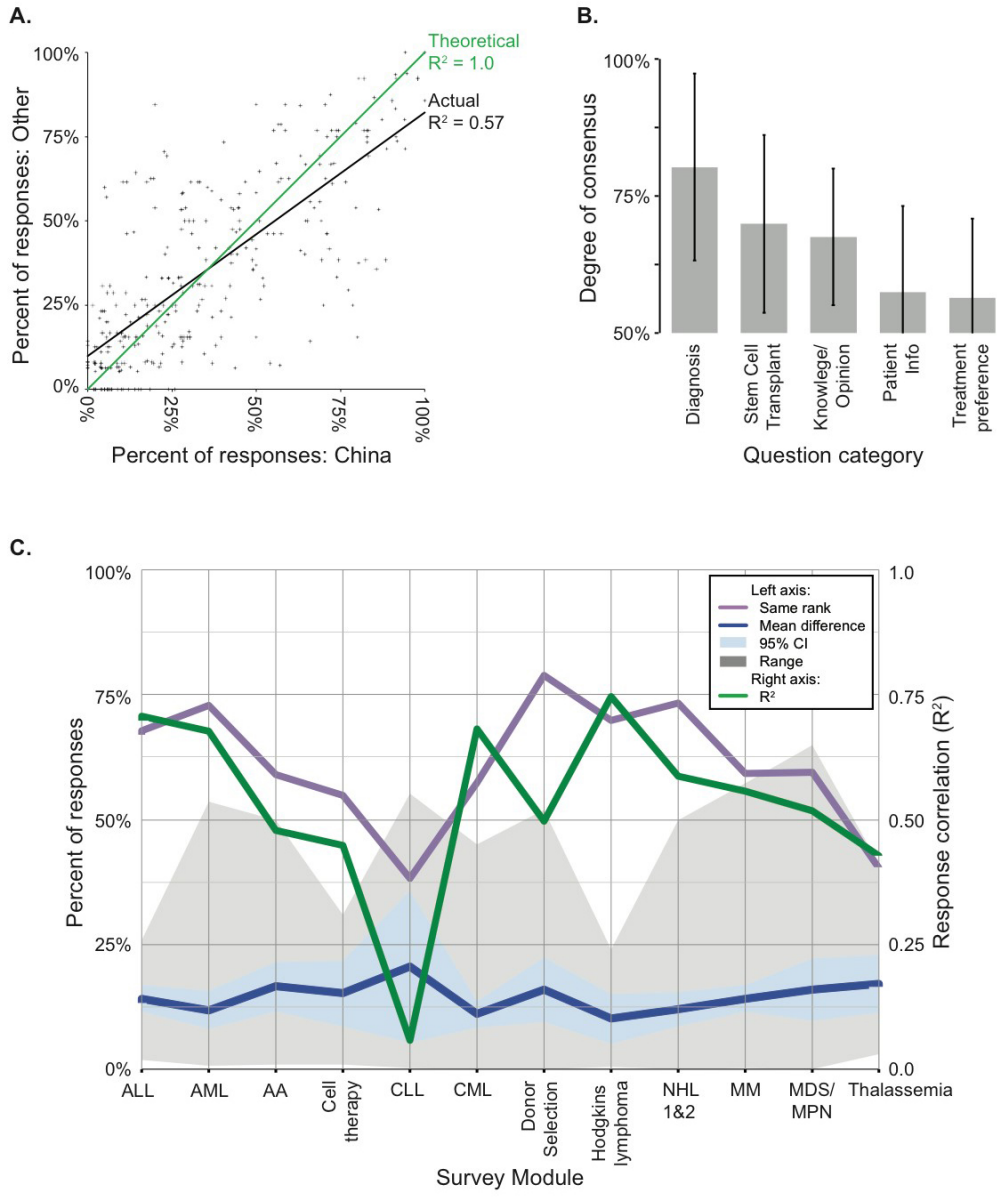
Meta analyses revealed varying levels of concordance between survey responses, depending on the national origin of the respondent (A), general category of question (B), or disease setting/topic (C) **A**. We plotted the percentage of responses by China (x-axis) and “Other” (y-axis) physicians for each answer choice across all survey questions (crosses), and calculated a linear best fit (R^2^, coefficient of determination, black line) as a measure of agreement between the geographic groups. A theoretical perfect 1:1 fit is represented by the green line. **B**. Each question was grouped into one of five general categories (x-axis), and the largest overall percent response among the answer choices captured as a proxy for the degree of consensus or unanimity. The average maximum response (or degree of consensus) is shown +/- 1 standard deviation. **C**. Numerous metrics were tabulated to describe the level of similarity between the stratified China and “Other” groups within and between each survey module (x-axis). The left vertical axis reports the percent of responses. The purple line indicates the percent of answer choices that were ranked in the same order between groups. The blue line represents the mean difference in percent response between China and Other for each answer choice, with 95% confidence intervals shaded light blue, and the absolute range in percent differences shaded grey. The linear best fit (R2) for each module was calculated as in (A), shown in green on the right vertical axis. CLL is an outlier due to only 2 questions, one of which was heavily skewed.

Overall geographical concordance in survey responses was positive and strong (Figure 1a). Questions regarding molecular or cytological diagnostics generally received similar percentages of responses between groups. When multiple diagnostic factors can be considered, physicians report considering most if not all of relevant indicators. For example, module for aplastic anemia (AA) asks: “Which investigations are routinely used to make a diagnosis of AA?” Most physicians (nearly 70%) selected all available answer choices, and the proportions based on nationality were identical to their representation in survey (χ^2^ *p*-value = 0.93). To give another example, in myelodysplastic/myeloproliferative module, we asked: “Do you perform JAK2 mutation analysis in all myeloproliferative disease?” Overall response was 97% yes, 3% no, with both geographic groups represented proportionally. We can conclude that this diagnostic approach has almost reached saturation among practitioners in our sample set. The NCCN guidelines also recommend to perform molecular testing for JAK2 V617F mutations in patients with suspicion of myeloproliferative neoplasms.

Perhaps more interesting than instances of absolute agreement, these data provide a unique opportunity to identify topics in which physician knowledge or opinions diverge. We assigned questions from all modules to broad categories of: patient info; diagnosis; treatment preference; transplantation; and general knowledge/opinion. We found that questions pertaining to treatment preference (i.e. drug choice) yielded least unanimous responses, on average, whereas diagnosis questions elicited the most unanimous choices (Figure 1b). For instance, Non-Hodgkin Lymphoma (NHL) module asked: “What is your treatment of choice for salvage therapy for NHL?” Here, proportional representation was nearly identical between geographic groups, but physicians did not report same approach to care. Half of all respondents chose “combination chemotherapy,” while other half were divided among “rituximab-lenalidomide,” “Bortezomib-lenalidomide-rituximab,” and “other.” Sheer number of questions with highly variable responses, especially in regards to treatment preference, indicates just how rarely we observed a single standard of care.

Examining geographic discrepancies also yielded an interesting perspective on what areas of treatment vary, and why. For example, chronic lymphocytic leukemia (CLL) module asked: “Your treatment of choice for relapsed/refractory CLL?” The majority of Chinese respondents selected Bendamustine (57%) with FCR second (38%). Meanwhile, 60% of the “Other” respondents chose Ibrutinib. Such a feedback is consistent with the existing NCCN guidelines where bendamustine +/- rituximab or reduced dose FCR are listed as options for relapsed/refractory CLL and Ibrutinib treatment is supported by category 1 level evidence.

Ibrutinib is a first-in-class non-chemotherapy tyrosine kinase inhibitor, but is not yet approved in China. Given the strong response we observed from “Other” group, one might predict the market impact this drug will have once it passes regulatory muster in China. Consider another example from donor selection module that asks: “What is your stem cell source of choice when donor options are limited?” Chinese respondents overwhelmingly chose haplo-identical donor (66%), while a majority of the “Other” group (57%) selected cord blood donor. Cord blood SCT is a relatively recent technology, with certain innovations and improvements just now emerging. Does newness of technology explain low adoption among Chinese physicians, do the data reflect a lack of practice in cord blood banking, or does some other barrier prevent this method from gaining prevalence?

We plotted several metrics to visualize overall concordance of responses between geographic groups for each module (Figure 1c). CLL was obvious outlier, but this may be discounted because the module contained only two questions, including ibrutinib example above. Thus one heavily weighted difference in drug availability is responsible for most of variation in CLL data. Nevertheless, for each module we calculated coefficient of determination (R^2^ linear best fit) between each answer choice for Other *versus* China (Figure 1c, green line). Highest R^2^ coefficient was observed for Hodgkin Lymphoma, ALL, CML and AML modules. Since all questions were multiple choice or simple yes/no, we tabulated percentage of responses that were chosen in same order, or rank, for each question. For nine modules, over half the responses were selected in same rank order (Figure 1c, purple line). Thus for most questions, geographic groups prioritized choices in same order, though not with same unanimity. We also asked how percentage of each geographic group selecting a given response varied. On average, we observed a geographic difference of 15% for any particular answer choice, and this was fairly constant across survey modules (Figure 1c, blue line). However, range of differences varied widely (Figure 1c, grey fill), suggesting some topics are subject to greater absolute disagreement.

### Leukemia

The main trend from this module was an overall high level of concordance between physicians from China and the other represented countries. In our final analysis we observed a coefficient of determination (R^2^) of 0.67 for all responses between these groups, and 68% of answers were chosen in the same rank order (Figure 1c). One topic of general agreement regarded the relatively new drug sorafenib, a tyrosine kinase inhibitor (TKI) that targets Fms-like tyrosine kinase 3 (FLT3). Most respondents (90% overall) would recommend allogeneic stem cell transplant for FLT3+ve AML in light of sorafenib availability which remains consistent with the NCCN guidelines, and 70% overall would employ sorafenib in post-transplant maintenance if FLT3 remains positive. However, nearly half of respondents (46%) in both groups expressed a similar desire for more data before they would adopt sorafenib for AML induction.

**Figure 2:**
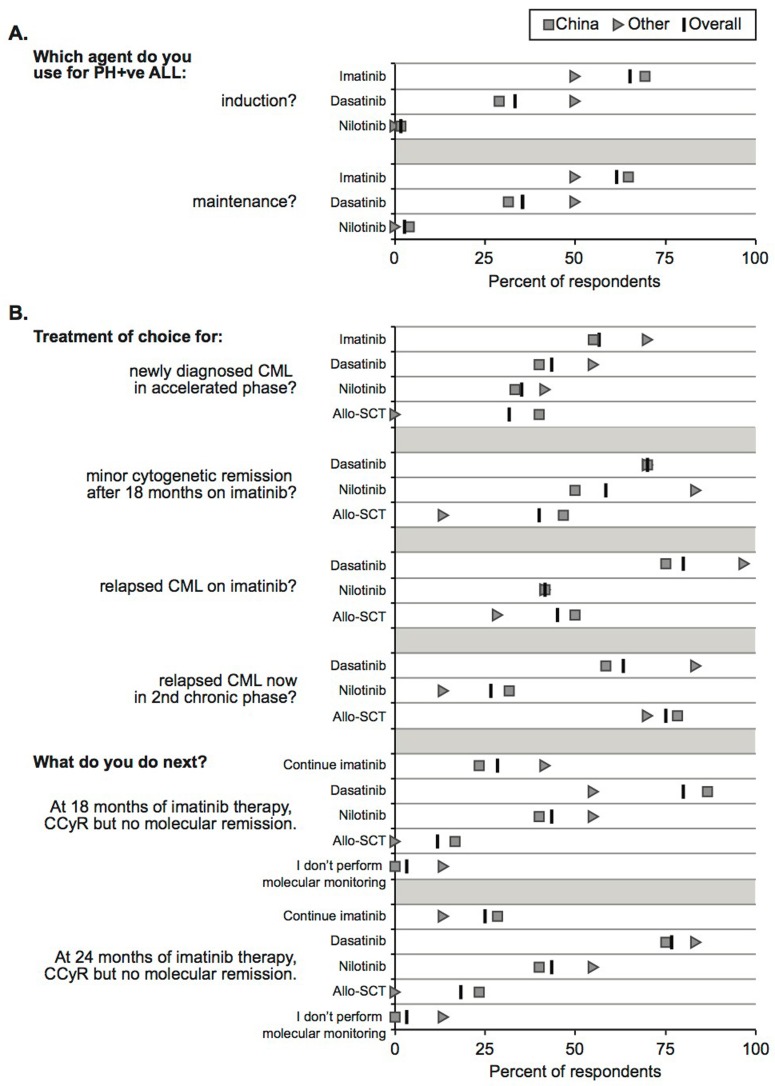

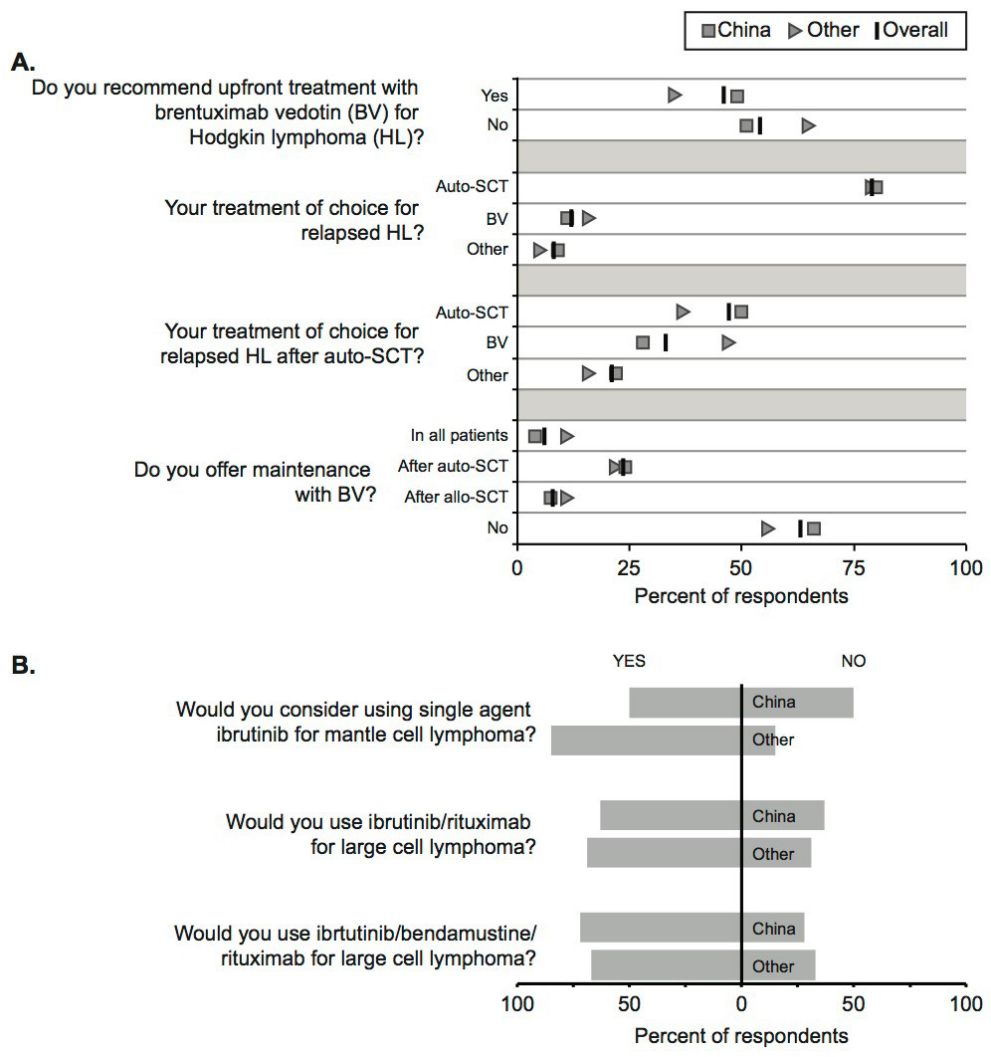
Physicians report different treatment preferences for acute lymphocytic leukemia (ALL, A) as well as for chronic myeloid leukemia (CML, B.) Here we display the results regarding the use of tyrosine kinase inhibitors imatinib, dasatinib and nilotinib. Each question is summarized to the left of the graph, with each answer choice immediately adjacent to the y-axis. As indicated in the legend above the figure, the percent of overall respondents (x-axis) that selected a particular answer choice is represented by a black bar. China and Other sub-groups are shown as squares and circles, respectively. Each question/answer set is separated by gray shading on the chart. Physicians reported treatment preferences for different scenarios regarding Hodgkin lymphoma (HL, C), as well as for non-Hodgkin lymphoma (NHL, D). **C**. The use of brentuximab vedotin (BV) varied depending on HL conditions among the two groups of respondents. Each question is summarized to the left of the graph, with each answer choice immediately adjacent to the y-axis. As indicated in the legend above the figure, the percent of overall respondents (x-axis) that selected a particular answer choice is represented by a black bar. China and Other sub-groups are shown as squares and circles, respectively. Each question/answer set is separated by gray shading on the chart. **D**. The China and Other groups’ intent to use ibrutinib varied between mantle cell and large cell lymphoma. Each question is provided to the left of the chart. The percent of physicians responding “Yes” (x-axis) is shown to the left of the vertical axis, while the “No” responses are shown to the right. Responses belonging to the geographic groups are labeled accordingly. **E**. Physicians from China were more likely to select stem cell transplant (SCT) from the multiple answer choices than were physicians in the Other group. The percent of respondents selecting each SCT answer choice are plotted with China on the y-axis and Other on the X-axis. The dotted line represents a 1:1 correspondence. The solid line represents the linear best-fit of the data, which is shifted upwards about 15 percentage points.

In the chronic myeloid leukemia (CML) module, we asked about treatment preferences under a series of disease conditions: newly diagnosed accelerated phase; minor cytogenetic remission after 18 months of imatinib-based therapy; relapse following imatinib therapy; relapse now in 2nd chronic phase; and complete cytogenetic response but no molecular response following 18 months of imatinib. For newly diagnosed CML, a plurality of all physicians preferred imatinib to dasatinib, nilotinib, or allo-SCT. Some consider imatinib the “gold standard” for first-line CML treatment, though others are wary due to a propensity for the development of resistance mutations during therapy [3–5]. All such options are also listed in the NCCN guidelines and the choice of the tyrosine kinase inhibitor (TKI) depends on the practicing physicians. In cases of relapse or remission following imatinib therapy, dasatinib was the plurality choice (Figure 2a, 2b). These drugs are all TKIs targeting the BCR-ABL fusion gene product, albeit with varying degrees of specificity and efficacy. Additional follow-up questions will be required to learn the reasons our respondents preferred one TKI over another in the same disease context. A larger percentage of the Chinese physicians selected allogenic stem cell transplant than did ‘Other’ cohort. This could reflect a difference in treatment philosophy, or a difference in relative availability/cost of drugs *versus* cell therapies [6].

Molecular testing and genomic profiling of acute lymphocytic leukemia (ALL) has featured prominently in literature. Physicians aim to determine the nature of the bone marrow microenvironment, the B- or T-cell lineage of the cancerous cells, and the specific mutations driving the neoplasm [7–9]. Consistent with these practice patterns, a large majority of physicians in our survey report performing molecular testing for ALL patients (89% China, 71% Other), and more still base their therapy decision on results from molecular testing (90% China, 86% Other). The difference in percentage between ‘Other’ cohort between these two responses may reflect workflows in which physicians do not themselves perform/order the testing, but rather see patients whom have already been tested. Alternatively, this discrepancy might be due to variation intrinsic in our response capture methods. Focused genetic testing and molecular screening of ALL patients aim to improve risk assessment, targeted therapy, and treatment outcomes. With these goals in mind, a large majority of survey respondents also perform minimal residual disease (MRD) analysis on all of their ALL patients (94% China, 71% Other). Therapy for ALL typically is dictated by the level of risk/aggressiveness of disease, best assessed with sensitive and fast MRD detection methods [10].

The survey included just two questions on Chronic Lymphocytic Leukemia (CLL). When asked about the treatment of choice for relapsed/refractory CLL, a majority of Chinese physicians selected bendamustine, an alkylating agent applied widely for CLL and other lymphomas. Meanwhile, a majority of non-Chinese respondents selected ibrutinib, a targeted inhibitor of Bruton's tyrosine kinase [11]. Ibrutinib is a cutting edge treatment gaining favor in the West and around the world, but at the time of the survey had not yet achieved regulatory approval in China. This simple difference in drug availability caused our CLL survey module to stand apart as an outlier in the above meta-analysis. Nevertheless, we believe these kinds of data are important for predicting the clinical penetration new pharmaceuticals may have upon regulatory approval and market availability.

### Lymphoma

The Hodgkin lymphoma (HL) module saw relatively high accord between Chinese and Other respondents (Figure 1c), though overall responses to any given question often varied widely (Figure 2c). For example, when asked whether one would recommend upfront treatment with brentuximab vedotin (BV) for Hodgkin lymphoma, 46% responded “yes” with 54% “no.” BV is approved in USA for patients in relapse following high-dose therapy, stem cell rescue, or multiple rounds of chemotherapy, but usually is not considered for upfront therapy [12, 13]. However, a recent phase II study, published after our survey was collected, demonstrated a benefit to front-line BV in older patients who would not tolerate typical chemotherapy regimes^13^. Autologous SCT was preferred for relapsed HL among 79% of respondents. Overall 68% of respondents offer allogenic SCT in relapse following auto-SCT. A majority (63%) do not offer BV maintenance therapy, though this too may be challenged by new information (Figure 2c). Another recent study makes a case for BV as maintenance after auto-SCT in high-risk patients [15]. We will be interested to document how physician attitudes towards BV therapy change over time as these and future clinical data become widely known.

Physicians in our survey frequently displayed strong agreement in regards to non-Hodgkin lymphomas. Answers regarding peripheral T-cell lymphoma, large cell lymphoma, salvage therapy, etc., were selected in similar proportions and in similar rank order between geographic groups. Even when asked ibrutinib, which was not available outside of clinical trials in China, respondents were consistent in their intent to use for large cell lymphoma (but not mantle cell lymphoma) (Figure 2d). Several questions did elicit strikingly different opinions along geographic lines, however. Whether to offer auto-SCT for high risk diffuse large B-cell lymphoma (DLBCL), Chinese physicians overwhelmingly said “yes” (80%), *versus* the international group's 38% “yes.” At this time high dose therapy is recommended for relapsed / refractory disease based on NCCN guidelines. Preferences for mantle cell lymphoma therapy also varied between these groups. The largest difference was the simple yes/no: Do you perform gene expression profiling on your patients? 86% of Chinese respondents were affirmative, *versus* 36% of the Others. For more than a decade gene microarrays have been used to classify subtypes of NHL, and with the eager adoption of next generation sequencing around the world, we predict that gene expression profiling will become a standard approach to pinpointing cancers and refining therapeutic approaches [16–18].

### Bone marrow dysfunction

Nearly all respondents in our survey treat del5q MDS patients with lenalidomide (95%), offer allo-SCT for high risk MDS (97%), and, as mentioned above, analyze JAK2 mutation status (97%). Despite a near unanimous concern over JAK2, 40% of Chinese respondents do not prescribe ruxolitinib, a novel JAK kinase inhibitor recently shown to be effective in the JAK positive MDS setting [19–21]. Newness of the ruxolitinib data suggest that future surveys will reveal a greater awareness and adoption of this treatment. A large majority of survey respondents (69%) offer induction prior to allo-SCT, most commonly with hypomethylating agents (HMA). However, HMA of choice varies between Chinese (80% decitabine) and Other physicians (85% 5-azacytidine), reflecting a difference in drug availability. Clinical studies and meta-analyses have sought to determine which therapy is most effective, concluding that while both HMAs are beneficial, 5-azacytidine has overall survival advantage [22–24].

Aplastic anemia (AA) module revealed several interesting geographic trends in patient composition and practice patterns. For instance, a large majority of Chinese physicians (88%) routinely use growth factors in the treatment of aplastic anemia, *versus* only 38% of the Other counterparts. Growth factors are sometimes used in patients who receive immunosuppressive therapy in lieu of SCT eligibility. Still, the value and efficacy of growth factors has been called into doubt by a number of primary studies and meta-analyses [25, 26]. We believe this common practice among our Chinese respondents reveals a need for updated, evidence-based protocols in the AA setting.

Though not every AA question yielded statistically different results between groups, we believe that various trends are worth additional attention in future studies. For example, when asked what criteria are used to determine transplant eligibility, most Chinese physicians selected all four choices: disease severity (82%), donor availability (82%), performance status (74%), and affordability (74%). Their international colleagues selected the first three of these at similar frequencies, but only 38% of the Others chose affordability as a criteria for transplant eligibility. This discrepancy may reflect the relative wealth of the patient population, the relative cost of treatment or availability of insurance coverage, and/or a more fiscally conservative approach to care delivery in the Chinese healthcare system.

Chinese physicians report seeing fewer thalassemia patients in general, and fewer high-risk class 3 patients in particular, than their international counterparts. Nevertheless, a greater percentage of Chinese respondents (72% *versus* 42% Other) offer allo-SCT to these high-risk patients. This is consistent with a general trend in the data across various cancer settings, whereby Chinese physicians are more likely to recommend allo-SCT, or to rank this treatment highly among the various options (Figure 2e).

The multiple myeloma (MM) module, by far the largest question set in our survey, has been recently published by Lu et al., [2] and thus we will not go into detail. Major take-away from the MM analysis is that Chinese physicians are eager to gain regulatory approval for new breakthrough therapies such as carfilzomib and elotuzumab, which have been shown to improve PFS after years of stagnation in treatment outcomes in this setting [27–29].

### Cell therapy

A majority of Chinese respondents (69%) perform cell therapy at their centers, *versus* 38% of Other physicians in the survey. Further, 96% of Chinese respondents perform post-transplant donor lymphocyte infusions in some scenarios, *versus* 69% of Others. When donor options are limited, 66% of Chinese respondents prefer a haplo-identical donor, while 57% of Other physicians recommend using cord blood. Cord blood as a stem cell source has emerged over the last half decade as a promising and effective alternative, but this technology clearly has not become a mainstay of the Chinese approach to transplantation [30]. Perhaps further research and advances in cell expansion, bone marrow targeting and GVHD minimization will lead to wider adoption of cord blood transplantation [31, 32]. Meanwhile, we observed similar patterns between both groups regarding CAR-T and NK cell therapy—a majority of physicians overall do not use either approach. Most Chinese respondents (71%), however, expressed optimism that CAR-T therapy will become routine in treating leukemia. Indeed, improving CAR-T efficacy and specificity is a major thrust of cancer immunotherapy research, with recent advances providing reason to be hopeful [33–35].

## CONCLUSIONS

Recognizing questions or entire disease settings in which answers vary between nations, or vary among physicians regardless of nationality, begins to address an age-old problem: How do we know what we do not know? This survey takes a big step towards illuminating gaps in access to or availability of therapeutic agents. These results reveal a utility and need for widespread and ongoing initiatives to assess knowledge and provide evidence-based education in real time. The entire global community of hematology-oncologists stands to benefit from contributing to a broad knowledge base. Data will be made all the more valuable by longitudinal participation, such that we can monitor changes in the state of the art over time.

By sharing our expertise, but also keeping an open mind to learn from those more expert than ourselves, we all stand to improve as physicians. And in doing so, the real winners will be patients, and that is why we practice in the first place.
